# Apparent Bilateral Aldosterone Suppression During Adrenal Vein Sampling: An Artefact of the Liaison Chemiluminescence Immunoassay and Other Causes Revealed by Comparisons With Another Commercial Immunoassay and Mass Spectrometry

**DOI:** 10.1111/cen.70135

**Published:** 2026-03-29

**Authors:** Christina Pamporaki, Carmina T. Fuss, Ralph Kickuth, Lydia Kürzinger, Mirko Peitzsch, Sybille Fuld, Manuel Schulze, Jun Yang, Martin Reincke, Sven Gruber, Felix Beuschlein, Jacques W. M. Lenders, Graeme Eisenhofer

**Affiliations:** ^1^ Department of Medicine III University Hospital Carl Gustav Carus, Technische Universität Dresden Dresden Germany; ^2^ Department of Internal Medicine I, Division of Endocrinology and Diabetes University Hospital, University of Würzburg Würzburg Germany; ^3^ Department of Radiology University Hospital, University of Würzburg Würzburg Germany; ^4^ Institute of Clinical Chemistry and Laboratory Medicine Medical Faculty and University Hospital Carl Gustav Carus, Technische Universität Dresden Germany; ^5^ Department Information Services and High Performance Computing, Center for Interdisciplinary Digital Sciences Technische Universität Dresden Germany; ^6^ Department of Medicine, Centre for Endocrinology and Reproductive Health, Hudson Institute of Medical Research Monash University Victoria Clayton Australia; ^7^ Department of Medicine IV University Hospital, Ludwig Maximilian University Munich Munich Germany; ^8^ Department of Endocrinology, Diabetology and Clinical Nutrition University Hospital Zurich and the LOOP Zurich Medical Research Center Zurich Switzerland; ^9^ Department of Internal Medicine Radboud University Medical Center Nijmegen Netherlands

**Keywords:** adrenal venous sampling, bilateral aldosterone suppression, immunoassay, mass spectrometry, primary aldosteronism

## Abstract

**Objective:**

Subtyping of primary aldosteronism usually requires adrenal vein sampling (AVS). Interpretation can be compromised by apparent bilateral aldosterone suppression (ABAS), which we hypothesised reflects an artefact of the Liaison immunoassay of aldosterone. We therefore compared measurements of aldosterone by liquid chromatography‐tandem mass spectrometry (LC‐MS/MS) with those by Liaison and iSYS immunoassays.

**Design:**

Observational multicentre study.

**Patients:**

The study involved 216 patients who underwent bilaterally selective non‐stimulated AVS.

**Measurements:**

Adrenal and peripheral venous plasma aldosterone concentrations were measured by Liaison and iSYS immunoassays in 110 and 106 respective samplings compared to LC‐MS/MS in all. Ratios of aldosterone‐to‐cortisol below 1.0 in adrenal versus peripheral vein samples defined relative aldosterone suppression.

**Results:**

Among all AVS procedures, 9.7% (21/216) of samplings with immunoassay measurements showed ABAS, threefold more (*p* = 0.0004) than the 3.2% (7/216) with LC‐MS/MS. Rates of ABAS were particularly high with the Liaison immunoassay compared to LC‐MS/MS (14.5% vs. 0.9%, *p* < 0.0001) and remained higher after substitution of immunoassay‐measured with LC‐MS/MS‐measured cortisol (10.9% vs. 0.9%, *p* = 0.0007). Among 106 procedures involving iSYS immunoassay measurements, rates of ABAS at 4.7% were a third (*p* = 0.0202) those of the Liaison immunoassay and did not differ from LC‐MS/MS measurements (5.7%). ABAS confined to LC‐MS/MS measurements in one patient was resolved by a second sampling that revealed pronounced aldosterone secretion versus earlier suppression.

**Conclusion:**

ABAS is a relatively common artefact of the Liaison immunoassay of aldosterone. More rarely, ABAS may reflect sampling blood from an adrenal venous tributary that does not drain from the site of excess aldosterone secretion.

## 1 Introduction

Adrenal vein sampling (AVS) is the recommended procedure for discriminating unilateral from bilateral subtypes of primary aldosteronism to thereby guide patients towards adrenalectomy versus medical management [1, 2]. Interpretation of results requires that catheters are correctly positioned in both adrenal veins, which is traditionally assessed from the ratio of cortisol concentrations in adrenal venous versus peripheral venous sampling sites. This ratio, termed the selectivity index, must be above a certain threshold, commonly ≥3 for cosyntropin‐stimulated sampling and ≥2 for non‐stimulated sampling [2]. Hypersecretion of aldosterone from one or both adrenals is then determined from the lateralisation index (LI), the ratio of aldosterone‐to‐cortisol in one adrenal vein compared to the other. A LI of ≥4 or ≥3, commonly used to respectively denote lateralisation for cosyntropin‐stimulated or non‐stimulated sampling, determines likelihood of unilateral disease to direct patients to surgical intervention [2].

Relative suppression of aldosterone secretion, reflected by an aldosterone‐to‐cortisol ratio in one adrenal vein lower than in a peripheral vein, may allow for additional interpretation of AVS results [1, 2]. The ratio has been termed the relative aldosterone secretion index (RASI). Contralateral suppression has been defined by a RASI < 1 in one adrenal vein when aldosterone secretion is lateralised to the other [3, 4]. Such suppression of aldosterone secretion is assumed to reflect regulatory impacts of the renin‐angiotensin‐aldosterone system in response to hypersecretion of aldosterone from the contralateral diseased adrenal and has been proposed to additionally support presence of lateralised disease [3, 4]. However, several studies have found no association between contralateral suppression and postsurgical outcomes [5, 6, 7, 8]. Nevertheless, when AVS is unilaterally selective but indicates suppressed aldosterone secretion, removal of the adrenal with non‐selective sampling has sometimes been justified on the basis of contralateral suppression. Reports of RASI values <1 in some patients with bilateral disease, however, highlight the need for caution with this approach. For both bilaterally selective and unilaterally selective sampling, interpretation may be improved by applying a more stringent RASI cut‐off of <0.5 [9, 10, 11, 12].

Further need for caution with use of the RASI to guide decision‐making has been indicated by findings of apparent bilateral suppression of aldosterone (ABAS) at prevalences from 9.5% to 25% [13, 14, 15, 16]. All reports involved immunoassay measurements; for two reports ABAS was resolved with mass spectrometry‐based measurements [15, 16]. A particularly high rate of ABAS (25%) was observed in a study that utilised the Liaison chemiluminescence immunoassay (CLIA) from Diasorin [16].

Based on observations of pronounced macromolecular likely protein‐based interferences with the Liaison immunoassay of aldosterone [17, 18], we hypothesised that the ABAS artefact observed by Wannachalee and colleagues [16] is due to the excessively poor accuracy of that particular immunoassay. To address this hypothesis, we reviewed data from 216 unstimulated bilaterally selective AVS procedures that allowed for paired comparisons of mass spectrometric with immunoassay data, including 110 measurements by the Liaison immunoassay and 106 with the iSYS immunoassay from Immuno Diagnostic Systems. We also examined post‐adrenalectomy outcomes in relation to bilateral and unilateral aldosterone suppression.

## 2 Materials and Methods

### 2.1 Patients

This multicentre study involved patients recruited into the prospective study on the diagnostic value of steroid profiling in primary aldosteronism (PROSALDO, registration no: DRKS00017084) according to inclusion and exclusion criteria outlined in the Supplemental Appendix. Between January 2019 and July 2025, a total of 1011 patients (522 females, 489 males, median age 50) provided written informed consent for their participation in the protocol, as approved by the ethics committees at all centres.

### 2.2 Study Procedures

Flow of patients through the protocol was according to study design features and clinical procedures as documented previously [17, 19, 20] and further detailed in Figure S1 and the associated text of the Supplemental Appendix. In brief, after use of the plasma aldosterone‐to‐renin ratio (ARR) and plasma steroid profiles to screen for PA, patients with positive results for either or both tests underwent the seated saline suppression test (SSST) or proceeded directly to subtyping when PA seemed clear. Subtyping according to anatomic imaging and AVS, and restricted to patients who agreed to the possibility of adrenalectomy, mostly followed positive results for the SSST. Blood samples during fluoroscopic guided non‐stimulated AVS were obtained simultaneously from each adrenal vein at all except one centre that employed sequential sampling and where matching peripheral vein samples were obtained for each sequential sample. Adrenalectomy was usually reserved for patients with bilaterally selective sampling and lateralisation to one adrenal, though in some patients relied on imaging evidence of a unilateral mass. Outcome assessments in patients who underwent adrenalectomy were undertaken to document biochemical and clinical remission as further detailed in the Supplemental Appendix.

### 2.3 Laboratory Measurements

Routine measurements of aldosterone throughout the protocol were by the Liaison CLIA (DiaSorin, Saluggia, Italy) at three centres, by the iSYS CLIA (Immuno Diagnostic Systems) at two centres and by liquid chromatography with tandem mass spectrometry (LC‐MS/MS) for the two Sydney centres. For this report, which focuses on immunoassay‐based measurements of aldosterone, data from the two Sydney centres were excluded (Figure S1). Measurements of cortisol for calculations of AVS selectivity, lateralisation and RASI values were by either the Liaison CLIA, the Elecsys electrochemiluminescence method (Roche Diagnostics, Base, Switzerland) or the Beckman Coulter‐based CLIA (Beckman Coulter Inc., Bria CA, USA) for the three centres that employed the Liaison CLIA to measure aldosterone. The two centres that employed the iSYS CLIA for aldosterone both used the Elecsys electrochemiluminescence method for measurements of cortisol. Renin for screening and at disease confirmation was measured by immunoassays as direct renin concentrations.

In addition to immunoasay measurements, additional specimens at each sampling time point were taken for measurements of plasma steroids by LC‐MS/MS according to a previously established method [21]. The steroid panel included aldosterone, cortisol, 11‐deoxycortisol, androstenedione and dehydroepiandrosterone (DHEA). The latter three steroids were employed as additional measures to cortisol for improved assessments of AVS selectivity [22]. Inter‐assay coefficients of variation at low/normal, mid and high plasma concentration ranges varied from 6.9% to 13.1% for aldosterone, 7.1% to 11.3% for cortisol, 6.2% to 8.7% for 11‐deoxycortisol, 7.9% to 13.8% for androstenedione and 13.6% to 24.2% for DHEA. Agreement of measurements obtained by LC‐MS/MS at Dresden and eight other centres established minimal bias of measurements [23, 24, 25].

### 2.4 Data Analyses

RASI values for right and left adrenal vein samplings were calculated according at an established equation in which the ratio for adrenal venous (AV) aldosterone to cortisol is divided by the ratio of peripheral venous (PV) aldosterone to cortisol:

$$\mathrm{RASI}=\;\frac{{\mathrm{AV}}_{\mathrm{aldosterone}}:{\mathrm{AV}}_{\mathrm{cortisol}}}{{\mathrm{PV}}_{\mathrm{aldosterone}}:{\mathrm{PV}}_{\mathrm{cortisol}}}$$

Skewed data were normalised by logarithmic transformation for purposes of display and use of parametric statistics. Statistical analyses were facilitated using the JMP Pro statistics software package version 18 (SAS Institute Inc., Cary, NC). Relationships between variables were examined using Pearson's or Spearman's correlation tests according to normal and non‐normal distributions of data. Comparisons of paired data, including LC‐MS/MS‐ versus immunoassay‐derived variables, utilised the Wilcoxon signed rank sum test. Comparisons of iSYS versus Liaison CLIA‐derived variables were by Chi‐square tests or construction of logistic regression models. Passing‐Bablok and Bland Altman analyses were used to examine differences in plasma concentrations of aldosterone and cortisol according to the different methods of measurement.

## 3 Results

### 3.1 Study Population

As outlined in the Supplemental Appendix, after screening and confirmatory tests there were 272 patients who underwent AVS (Figure S1). Among those there were 216 patients in whom both immunoassay and LC‐MS/MS data were available and in whom AVS was bilaterally selective according to selectivity indices ≥2 for at least three of four steroids (cortisol, 11‐deoxycortisol, androstenedione and DHEA). The study population included 94 females and 122 males with a median age of 51 years (range: 22–75). Immunoassay measurements of aldosterone were by the Liaison CLIA in 106 patients and by the iSYS CLIA in 110 patients.

### 3.2 Apparent Bilateral Aldosterone Suppression

**Figure 1:**
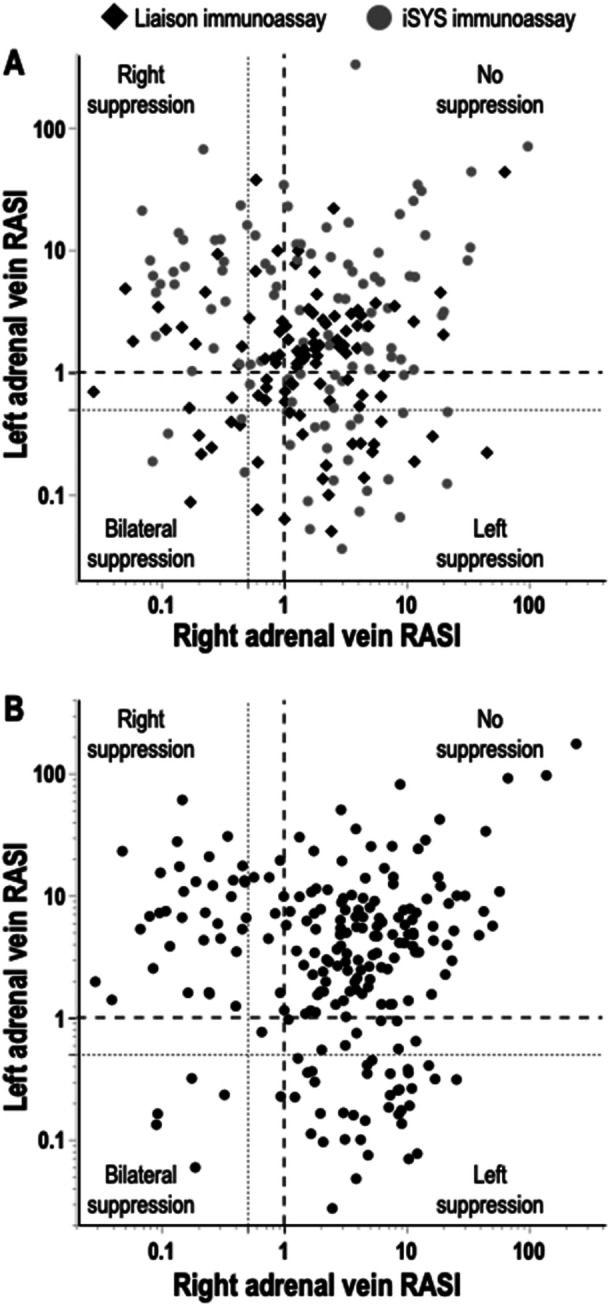
Scatter plots of right versus left adrenal vein RASI values for Liaison and iSYS immunoassay‐based measurements (A) and LC‐MS/MS‐based measurements (B) of aldosterone and cortisol. Dashed and dotted lines depict respective RASI cut‐offs of ≤1 and ≤0.5 that suggest suppression of aldosterone secretion. Different symbols in panel A represent measurements by the Liaison (◆) and iSYS (●) immunoassays.

Scatterplots of RASI values for right versus left adrenal vein samplings and measurements by immunoassays revealed 21 out of 216 patients in whom RASI values were <1 for both adrenal veins (Figure 1A). In contrast there were only 7 out of 216 patients in whom RASI values were <1 for both sampling sites with LC‐MS/MS measured aldosterone and cortisol (Figure 1B). Paired comparisons indicated a threefold higher (*p* = 0.0004) prevalence of ABAS with immunoassay than with LC‐MS/MS measurements (9.7% vs. 3.2%). At a cut‐off for the RASI of 0.5, the prevalence of ABAS remained higher (*p* = 0.0250) for immunoassay than LC‐MS/MS measurements (4.6% vs. 2.3%).

Six of the seven patients with ABAS according to LC‐MS/MS measurements also had ABAS according to immunoassay measurements while one patient (#22) had ABAS confined to LC‐MS/MS measurements (Table 1). AVS was bilaterally selective in all patients with ABAS according to selectivity indices ≥2 for at least three of the four steroids used to confirm catheter placement (Table S1). Plasma concentrations of aldosterone were on average measured 2.2‐fold higher (*p *< 0.0001) by immunoassays than by LC‐MS/MS in peripheral veins but showed no differences in adrenal veins (Table S2).

**Table 1:** RASI values for right and left adrenal vein samplings according to measurements of aldosterone and cortisol by immunoassay versus LC‐MS/MS methods in 22 patients with apparentf bilateral aldosterone suppression.

Patient	Immunoassay method		Immunoassay		LC‐MS/MS	
	Aldosterone	Cortisol	Right	Left	Right	Left
1	Liaison	Elecsys	0.437	0.370	1.878	1.536
2	Liaison	Elecsys	0.425	0.394	3.956	2.108
3	Liaison	Elecsys	0.028	0.693	0.029	1.966
4	Liaison	Elecsys	0.616	0.647	4.359	5.413
5	Liaison	Liaison	0.256	0.243	1.004	1.146
6	Liaison	Liaison	0.202	0.305	2.703	3.625
7	Liaison	Elecsys	0.168	0.512	0.164	1.596
8^a^	Liaison	Elecsys	0.210	0.215	0.326	0.233
9	Liaison	Beckman	0.172	0.087	1.979	0.164
10	Liaison	Elecsys	0.602	0.075	1.658	0.112
11	Liaison	Liaison	0.610	0.183	3.072	0.611
12	Liaison	Liaison	0.706	0.593	4.020	2.957
13	Liaison	Liaison	0.721	0.872	3.179	2.859
14	Liaison	Elecsys	0.716	0.848	2.078	2.375
15	Liaison	Liaison	0.377	0.620	1.446	2.689
16	Liaison	Elecsys	0.948	0.693	0.950	1.915
17^a^	iSYS	Elecsys	0.526	0.797	0.657	0.761
18^a^	iSYS	Elecsys	0.113	0.316	0.093	0.163
19^a^	iSYS	Elecsys	0.449	0.415	0.176	0.318
20^a^	iSYS	Elecsys	0.092	0.179	0.091	0.118
21^a^	iSYS	Elecsys	0.476	0.152	0.188	0.059
22^b^	iSYS	Elecsys	2.552	0.515	0.940	0.226

a Patients with evidence of bilateral aldosterone suppression by both immunoassay and LC‐MS/MS measurements.

b Patient 22 is the sole patient with bilateral suppression by LC‐MS/MS and not by immunoassay measurements.

ABAS was threefold more prevalent (*p* = 0.0202) with the Liaison CLIA (16/110) than with iSYS CLIA (5/106) measurements of aldosterone (Figure 1A and Table 1). Paired comparisons indicated a 16‐fold higher (*p* < 0.0001) prevalence of ABAS with Liaison CLIA than with LC‐MS/MS measurements of aldosterone (14.5% vs. 0.9%). At a lower cut‐off for the RASI of 0.5, differences in prevalence of ABAS remained significant (*p* = 0.0247) at 5.5% and 0.9% for aldosterone measured by the Liaison CLIA versus LC‐MS/MS. In contrast, prevalences of ABAS according to measurements of aldosterone by the iSYS CLIA and LC‐MS/MS did not differ at cut‐offs for the RASI of <1 (4.7% vs. 5.7%) and <0.5 (3.7% vs. 3.7%).

To determine whether the above differences in ABAS reflected impacts of immunoassay measurements of cortisol, RASI values were recalculated using immunoassay measurements of aldosterone and LC‐MS/MS measurements of cortisol (Figure S2). With that recalculation, the prevalence of ABAS remained specifically higher (*p* = 0.0007) for measurements of aldosterone by the Liaison CLIA than by LC‐MS/MS (10.9% vs. 0.9%) and did not differ according to measurements of aldosterone by the iSYS CLIA and LC‐MS/MS (4.7% vs. 5.7%).

### 3.3 Subtyping, Adrenalectomy and Post‐Surgical Outcomes in Patients With ABAS

Among all 22 patients with ABAS, 7 showed evidence of lateralised aldosterone secretion, including 6 by both immunoassay and LC‐MS/MS measurements and a 7th (#9) by LC‐MS/MS alone (Table 2). There was a trend to higher (*p* = 0.0574) values for lateralisation indices by LC‐MS/MS than by immunoassay measurements, which became significant (*p* = 0.0045) after comparisons were confined to the 16 patients (1–16) with ABAS indicated by the Liaison immunoassay.

**Table 2:** Presence of apparent bilateral aldosterone suppression (ABAS) by immunoassay (IA) and LC‐MS/MS measurements, AVS lateralisation, imaging evidence of disease and adrenalectomy (ADX) with outcomes.

Patient	Bilateral suppression		Lateralised	Lateralisation index		Imaging	ADX/Cure
	IA	LC‐MS/MS	Adrenal	IA	LC‐MS/MS	Findings	
1	Yes	No	Neither	1.2	1.2	No mass	No/No
2	Yes	No	Neither	1.1	1.9	No mass	No/No
3	Yes	No	Left	25.0	68.5	Left	Yes/Yes
4	Yes	No	Neither	1.1	1.2	Left	No/No
5	Yes	No	Neither	1.0	1.1	No mass	No/No
6	Yes	No	Neither	1.5	1.3	No mass	No/No
7	Yes	No	Left	3.0	9.7	No mass	No/No
8	Yes	Yes	Neither	1.0	1.4	Left	No/No
9	Yes	No	Right	2.0	12.1	No mass	No/No
10	Yes	No	Right	8.0	14.9	Right	Yes/Yes
11	Yes	No	Right	3.3	5.0	No mass	No/No
12	Yes	No	Neither	1.2	1.4	No mass	No/No
13	Yes	No	Neither	1.2	0.9	No mass	No/No
14	Yes	No	Neither	1.2	1.1	No mass	No/No
15	Yes	No	Neither	1.6	1.9	Left	No/No
16	Yes	No	Neither	0.7	2.0	Left	No/No
17	Yes	Yes	Neither	1.5	1.2	No mass	No/No
18	Yes	Yes	Neither	2.8	1.8	Bilateral	No/No
19	Yes	Yes	Neither	0.9	1.8	Bilateral	No/No
20	Yes	Yes	Neither	1.9	1.3	Bilateral	No/No
21	Yes	Yes	Right	3.1	3.2	Bilateral	No/No
22^a^	No	Yes	Right	5.0	4.2	No mass	Yes/Yes

*Note:* Patients 1–16 are those who showed ABAS according to the Liaison CLIA, whereas patients 17‐21 are those who showed ABAS according to the iSYS CLIA (see Table 1).

a Patient 22 is the sole patient who presented with ABAS by LC‐MS/MS and not by immunoassay measurements and in whom additional right adrenal vein sampling indicated an increased right‐sided lateralisation index of 98.3 and no suppression, which then justified a right adrenalectomy.

Adrenal imaging indicated left adrenal masses in five patients, including one (#3) who showed pronounced left‐sided lateralisation and who underwent a left adrenalectomy with subsequent normalisation of biochemistry and blood pressure (Table 2). A right adrenal adenoma was indicated by imaging studies in another patient (#10) who showed right‐sided lateralisation and who also showed post‐adrenalectomy biochemical and clinical remission.

Twelve of the 22 patients with ABAS showed no imaging evidence of an adrenal mass while 4 had bilateral adrenal lesions (Table 2). Among the former group there was a single patient (#22) with ABAS by LC‐MS/MS but not immunoassay measurements. Although this patient showed limited right‐sided lateralisation, a second sampling of the right adrenal vein revealed 55‐fold higher plasma concentrations of aldosterone (49,717 vs. 907 pmol/L) and a right adrenal lateralisation index of 98.3 that justified adrenalectomy. At post‐adrenalectomy outcome assessment, the patient showed normalised biochemistry though blood pressure remained elevated.

### 3.4 Post‐Surgical Outcomes According to Contralateral Suppression and Assay Method

Among all 216 patients, comparisons of patients with contralateral aldosterone suppression to those with ABAS or no suppression of aldosterone indicated differing proportions of patients who underwent adrenalectomy and showed biochemical remission dependent on the assay method (Table 3). Agreement about presence of contralateral suppression, bilateral suppression or no suppression of aldosterone reached 90% for measurements by LC‐MS/MS and the iSYS CLIA. That agreement was higher (*p* = 0.0045) than the 75% agreement between measurements by LC‐MS/MS and the Liaison CLIA, the latter method more frequently indicating contralateral suppression than indicated by LC‐MS/MS (45% *vs.* 31%). That increased frequency of contralateral aldosterone suppression with the Liaison CLIA was accompanied by a lowered association to patients who showed biochemical remission. Thus, pairwise comparisons showed stronger (*p* = 0.0022) agreement of contralateral suppression with biochemical remission according to measurements by LC‐MS/MS than by the Liaison CLIA. In contrast, there was no difference (*p* = 0.7646) in agreement between measurements by LC‐MS/MS and the ISYS CLIA.

**Table 3:** Adrenalectomy and post‐adrenalectomy outcomes according to presence or absence of contralateral aldosterone suppression estimated from results of Liaison and iSYS immunoassays (IA) versus LC‐MS/MS.

		Adrenalectomy		Biochemical remission^a^	
		Yes	No	Yes	No^a^
**Liaison IA vs. LC‐MS/MS**		***n* = 29**	***n* = 81**	***n* = 25**	***n* = 85** a
Contralateral suppression^b^					
Liaison IA	*n* = 50	46% (23/50)	54% (27/50)	38% (19/50)	62% (31/50)
LC‐MS/MS	*n* = 34	68% (23/34)	32% (11/34)	56% (19/34)	44% (15/34)
No contralateral suppression^b^					
Liaison IA	*n* = 60	10% (6/60)	90% (54/60)	10% (6/60)	90% (54/60)
LC‐MS/MS	*n* = 76	8% (6/76)	92% (70/76)	8% (6/76)	92% (70/76)
**iSYS IA versus LC‐MS/MS**		***n* = 54**	***n* = 52**	***n *= 44**	***n *= 62** a
Contralateral suppression^b^					
iSYS IA	*n* = 55	80% (44/55)	20% (10/55)	65% (36/55)	35% (19/55)
LC‐MS/MS	*n* = 50	84% (42/50)	16% (8/50)	66% (33/50)	34% (17/50)
No contralateral suppression^b^					
iSYS IA	*n* = 51	20% (10/51)	80% (42/51)	16% (8/51)	84% (43/51)
LC‐MS/MS	*n* = 56	21% (12/56)	79% (44/56)	20% (11/56)	80% (45/56)

a Total numbers of patients without evidence of biochemical remission include the 133 patients who did not undergo adrenalectomy, 11 who underwent adrenalectomy and did not reach outcome assessment, and 3 who did not show post‐adrenalectomy biochemical cure.

b Contralateral suppression was defined as a RASI value ≤1 in one adrenal vein, whereas no contralateral suppression was defined as either RASI values ≤1 in both adrenal veins (i.e., ABAS) or RASI values >1 in both adrenal veins (see Figure 1 for explanation).

### 3.5 RASI Values for Right and Left Adrenal Vein Sampling

**Figure 2:**
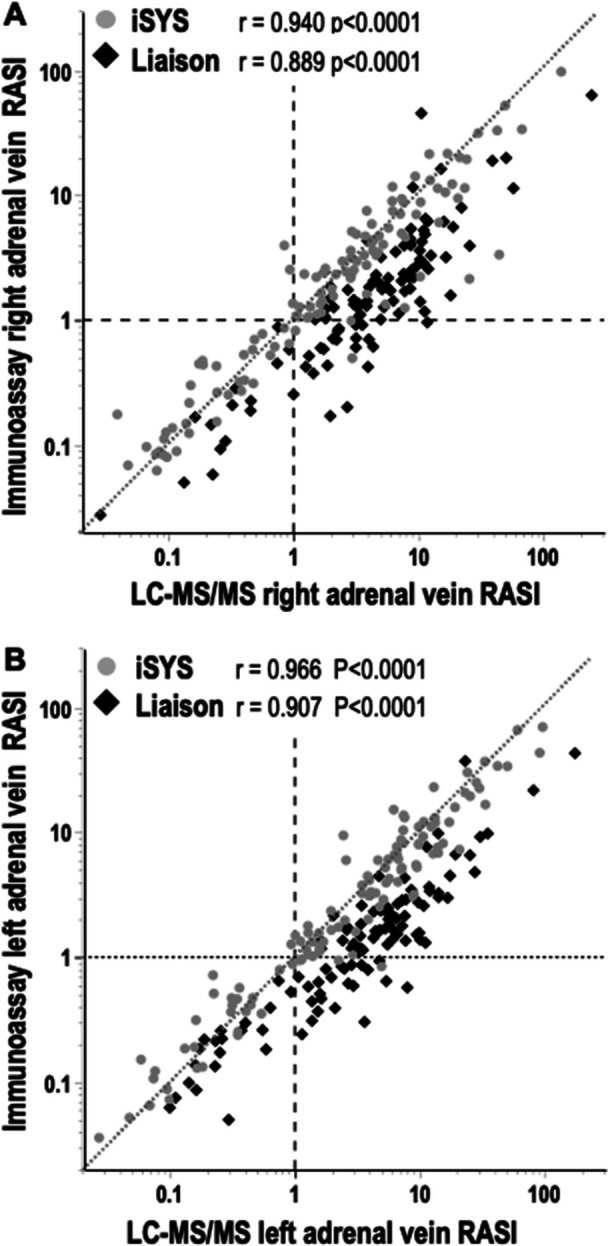
Scatter plot relationships of RASI values calculated using immunoassay‐based measurements (*y*‐axes) versus LC‐MS/MS‐based measurements (*x*‐axes) for right (A) and left (B) adrenal veins. Dashed lines depict RASI cut‐offs of ≤1 that suggest suppression of aldosterone secretion.

RASI values for samplings of right and left adrenal veins calculated using LC‐MS/MS measured aldosterone and cortisol displayed strong positive relationships (*p *< 0.0001) with both iSYS and Liaison CLIA‐based measurements of aldosterone (Figure 2). Relationships of RASI values calculated using the iSYS CLIA showed reasonable agreement around the line of identity to values calculated by LC‐MS/MS. More specifically, RASI values based on LC‐MS/MS and iSYS immunoassay measurements of aldosterone did not differ significantly for right adrenal vein samplings, though were 9% lower (*p* = 0.0283) by the iSYS CLIA than by LC‐MS/MS measurements for left adrenal vein samplings.

In contrast to the above observations, RASI values were 63% and 66% lower (*p *< 0.0001) for the Liaison CLIA than LC‐MS/MS measurements of aldosterone for respective right and left samplings (Figure 2). Thus, compared to the negligible shift in RASI values for measurements by the iSYS CLIA versus LC‐MS/MS, there was a considerably larger (*p *< 0.0001) shift for RASI values with measurements by the Liaison CLIA. The shift remained significant after substitution of LC‐MS/MS measured cortisol in calculated RASI values (Figure S3). Specifically, RASI values obtained with the Liaison CLIA were nearly 50% lower (*p* < 0.000) than those derived with LC‐MS/MS.

### 3.6 Immunoassay Versus LC‐MS/MS Measured Aldosterone and Cortisol

**Figure 3:**
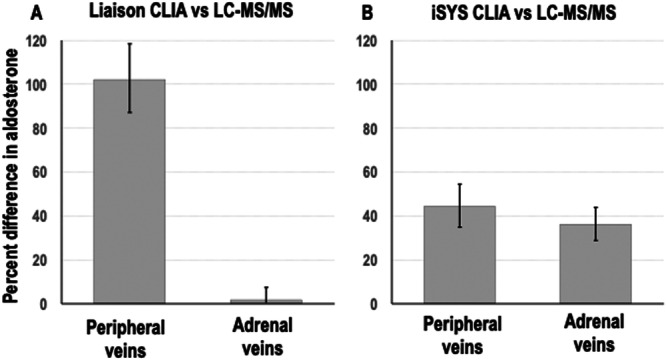
Percent differences in measured concentrations of aldosterone by Liaison (A) and iSYS (B) immunoassays compared to measurements by LC‐MS/MS for peripheral vein compared to adrenal vein samples. Results are shown as geometric means with confidence intervals.

Strong positive relationships (*p* < 0.0001) of immunoassay and LC‐MS/MS measured plasma aldosterone were observed for both Liaison and iSYS CLIAs (Figure S4). Although Bland‐Altman analyses indicated similarly minimal 28% and 39% higher (*p *< 0.0001) measurements of aldosterone by respective Liaison and iSYS immunoassays compared to LC‐MS/MS, those differences diverged at low and high plasma concentrations. Whereas plasma concentrations of aldosterone measured by the iSYS CLIA were higher than by LC‐MS/MS over all concentration ranges, the differences for the Liaison CLIA were only manifest at lower concentrations. Peripheral venous plasma concentrations of aldosterone were thus 102% higher according to measurements by the Liaison CLIA compared to LC‐MS/MS, but not different in adrenal venous plasma (Figure 3). In contrast, measurements by the iSYS CLIA were 44% and 36% higher than measurements by LC‐MS/MS in respective peripheral and adrenal venous samples.

As detailed in Figure S4, measurements of cortisol by immunoassays showed strong agreement with measurements by LC‐MS/MS. Agreement was particularly strong for the Elecsys immunoassay used at three of the five centres, which returned measured concentrations of cortisol only 10% higher than those measured by LC‐MS/MS. In contrast, Liaison and Bechman immunoassay‐based measurements of cortisol were 43% to 46% higher than by LC‐MS/MS.

## 4 Discussion

This study builds on two recent reports that documented unprecedented high prevalences of ABAS associated with immunoassay measurements that were resolved by LC‐MS/MS measurements [15, 16]. In the study by Wannachalee et al. [16], which involved non‐stimulated sampling followed by stimulated sampling, the overall prevalence of ABAS was 25% and ranged from 17.6% and 13.4% in respective stimulated and non‐stimulated samplings. Similarly, Li et al. observed an ABAS prevalence of 10.7% by immunoassay versus only 3.1% by LC‐MS/MS with non‐stimulated sampling [15]. The latter aligns closely with the 3.2% prevalence we observed using LC‐MS/MS. The higher prevalences of ABAS with immunoassays are consistent with our findings of 14.5% prevalence with the Liaison immunoassay, the same assay used in the two earlier studies. However, neither study definitively linked the artefact to the specific immunoassay; instead, inaccuracies in the measurement of both aldosterone and cortisol were cited as potential contributors.

The present report resolves the above issues by establishing that it is inaccuracy of the Liaison immunoassay from Diasorin that is the primary cause of ABAS, though our data also indicate involvement of other factors for the lower rates of ABAS with aldosterone measured by LC‐MS/MS and the iSYS immunoassay. Clarification that the Liaison immunoassay of aldosterone is at fault was indicated by the data from iSYS immunoassay versus LC‐MS/MS measurements and the retained high prevalence of ABAS with the Liaison immunoassay after replacement of immunoassay‐measured with LC‐MS/MS‐measured cortisol. Finally, Passing‐Bablok and Bland‐Altman analyses revealed that RASI values were underestimated for the Liaison immunoassay due to overestimation of plasma aldosterone confined to lower concentrations, particularly in peripheral venous samples.

That the ABAS artefact primarily reflects a problem with the Liaison immunoassay of aldosterone was predicted by previous observations that this assay suffers from particularly severe and patient‐variable over‐estimation of aldosterone at low concentrations during the SSST [17]. The resulting high prevalence of false‐positive results for the SSST, together with considerable patient‐to‐patient variability, indicates limited value of the Liaison CLIA for confirmatory testing. As described elsewhere [17, 18] and covered in the final section of the supplement, re‐assay‐after‐purification studies established that macromolecular interferences, most likely protein‐based, are the cause of the immunoassay inaccuracy. Cross‐reactivity with other steroids or similarly structured small molecules was ruled out to cause the interference. Although the specific proteins responsible for the interference have not been identified, there are numerous potential candidates [26, 27, 28, 29].

The same protein‐based and patient‐variable interferences that confound use of the Liaison CLIA for confirmatory testing likely also compromise use of that assay for interpretation of AVS studies. Our findings that ABAS varies according to the particular immunoassay is consistent with previous observations that compromised performance of the SSST also varies according to the immunoassay [17], and likely also explains different prevalences of ABAS in past studies. In a study of 1397 patients who underwent non‐stimulated AVS over a 34‐year period, only 2.6% displayed ABAS according to radioimmunoassay measurements of aldosterone [30]. On the other hand, two studies from Japan that employed different locally available radioimmunoassays reported 9.5% and 18.0% prevalences of ABAS with non‐stimulated AVS [13, 14].

Aside from interferences with immunoassay measurements, our data indicate other causes for ABAS. In particular, findings of ABAS in one patient in whom a subsequent right adrenal vein sampling indicated 55‐fold higher plasma concentrations of aldosterone and strong right‐sided lateralized aldosterone secretion indicate that differences in placement of catheters can also lead to findings of ABAS. Anatomical variations in adrenal vein drainage are well established [31, 32, 33]. Several case reports have clarified how such variants can result in ABAS and potentially confuse interpretation of AVS studies [34, 35, 36, 37, 38]. Among these cases, Zelinka et al. proposed that ABAS with discrepant right‐sided aldosterone suppression most likely resulted from positioning of the sampling catheter in a right adrenal vein tributary that did not drain from the diseased part of the adrenal [34]. Four cases of ABAS among a series of 61 patients have also been described, including three in which ABAS was resolved after repeated AVS that showed right‐sided lateralised aldosterone secretion that as in our patient then led to curative adrenalectomy [39]. These authors also proposed that ABAS likely resulted from super‐selective right adrenal venous sampling. Similarly, in our patient in whom bilaterally selective sampling of the right adrenal vein was performed twice in the same procedure, it seems that ABAS may have resulted from placement of the sampling tip of the catheter in an adrenal vein tributary that did not drain from the site of the aldosterone‐producing adenoma.

Considerations that ABAS can result from variable placement of sampling catheters within adrenal veins raise the possibility that such effects might also contribute to apparent bilateral disease in patients with unilateral disease in whom sampling from the diseased adrenal does not fully capture the source of excess aldosterone. Such cases have been indicated in the study of Zelinka et al. [34]. More recently other cases that might reflect the same problem have been indicated by comparisons of ^11^C‐metomidate functional imaging with AVS for subtyping patients with primary aldosteronism [40].

Apart from the ABAS artefact, this study also indicates that inaccurate measurements of aldosterone by the Liaison CLIA obscures associations of contralateral aldosterone suppression with successful post‐adrenalectomy outcomes. Such inaccuracies may have confounded interpretations of previous studies suggesting that contralateral aldosterone suppression is not predictive of successful post‐surgical outcomes [5, 6, 7]. On the other hand, over‐steering of catheters in super‐selective sampling could conceivably also result in apparent contralateral aldosterone suppression, which might obscure presence of unilateral disease or even possibly mislead clinicians to presence of disease in the wrong adrenal.

In conclusion, ABAS occurs commonly as an artefact of severe patient‐variable interferences with Liaison CLIA‐based measurements of aldosterone. Less commonly ABAS may occur from other causes. Given the considerable expense and effort that must be expended in AVS studies, it might be considered that the most appropriate analytical methods should be employed to guide clinical decision‐making. For those centres that employ the Liaison CLIA to measure aldosterone, accumulating evidence now supports discontinuing that assay. Increasing availability of LC‐MS/MS makes this more widely possible than before. Improved agreement of immunoassay with LC‐MS/MS measurements may also be reached with a new generation of CLIAs that employ non‐competitive sandwich technology [41, 42]. Such emerging technologies if suitably validated may offer alternative solutions.

## Supporting information

Supplement‐Bilateral‐suppression‐26122025.

## Data Availability

The data that support the findings of this study are available from the corresponding author upon reasonable request.

## References

[1] S. Monticone , A. Viola , D. Rossato , et al., “Adrenal Vein Sampling in Primary Aldosteronism: Towards a Standardised Protocol,” The Lancet Diabetes & Endocrinology 3, no. 4 (2015): 296–303. 24831990 10.1016/S2213-8587(14)70069-5

[2] G. P. Rossi , M. Battistel , T. M. Seccia , F. B. Rossi , and G. Rossitto , “Subtyping of Primary Aldosteronism by Adrenal Venous Sampling,” Endocrine Reviews 46, no. 4 (2025): 501–517. 39965116 10.1210/endrev/bnaf007 PMC12259235

[3] M. J. Wolley , R. D. Gordon , A. H. Ahmed , and M. Stowasser , “Does Contralateral Suppression at Adrenal Venous Sampling Predict Outcome Following Unilateral Adrenalectomy for Primary Aldosteronism? A Retrospective Study,” The Journal of Clinical Endocrinology & Metabolism 100, no. 4 (2015): 1477–1484. 25636049 10.1210/jc.2014-3676

[4] H. Umakoshi , K. Tanase‐Nakao , N. Wada , et al., “Importance of Contralateral Aldosterone Suppression During Adrenal Vein Sampling in the Subtype Evaluation of Primary Aldosteronism,” Clinical Endocrinology 83, no. 4 (2015): 462–467. 25727719 10.1111/cen.12761

[5] S. Monticone , F. Satoh , A. Viola , et al., “Aldosterone Suppression on Contralateral Adrenal During Adrenal Vein Sampling Does Not Predict Blood Pressure Response After Adrenalectomy,” The Journal of Clinical Endocrinology & Metabolism 99, no. 11 (2014): 4158–4166. 25119314 10.1210/jc.2014-2345

[6] M. Tagawa , M. Ghosn , H. Wachtel , et al., “Lateralization Index But Not Contralateral Suppression at Adrenal Vein Sampling Predicts Improvement in Blood Pressure After Adrenalectomy for Primary Aldosteronism,” Journal of Human Hypertension 31, no. 7 (2017): 444–449. 28079049 10.1038/jhh.2016.92

[7] D. A. Dominguez , P. Chatani , R. Murphy , et al., “Contralateral Suppression Index Does Not Predict Clinical Cure in Patients Undergoing Surgery for Primary Aldosteronism,” Annals of Surgical Oncology 28, no. 12 (2021): 7487–7495. 33939050 10.1245/s10434-021-09692-7 PMC8530859

[8] S. Mørup , N. Voss , C. Clausen , C. L. Feltoft , M. Andreassen , and J. Krogh , “Prognostic Value of Contralateral Suppression for Remission After Surgery in Patients With Primary Aldosteronism,” Clinical Endocrinology 96, no. 6 (2022): 793–802. 35060161 10.1111/cen.14678

[9] V. Strajina , Z. Al‐Hilli , J. C. Andrews , et al., “Primary Aldosteronism: Making Sense of Partial Data Sets From Failed Adrenal Venous Sampling‐Suppression of Adrenal Aldosterone Production Can Be Used in Clinical Decision Making,” Surgery 163, no. 4 (2018): 801–806. 29174432 10.1016/j.surg.2017.10.012

[10] O. Suntornlohanakul , S. Soonthornpun , W. Srisintorn , R. D. Murray , and N. Kietsiriroje , “Performance of the Unilateral Av/Ivc Index in Primary Hyperaldosteronism Subtype Prediction: A Validation Study in a Single Tertiary Centre,” Clinical Endocrinology 93, no. 2 (2020): 111–118. 32347973 10.1111/cen.14210

[11] J. Okubo , P. Frudit , A. C. B. S. Cavalcante , et al., “Contralateral Suppression in Adrenal Venous Sampling Predicts Clinical and Biochemical Outcome in Primary Aldosteronism,” The Journal of Clinical Endocrinology & Metabolism 109, no. 9 (2024): 2282–2293. 38442744 10.1210/clinem/dgae142

[12] C. M. T. Chow , M. S. C. Lai , X. Lo , and Y. W. S. Liu , “Role of Unilateral‐Cannulating Adrenal Venous Sampling for the Subtyping of Primary Aldosteronism for Adrenalectomy: Experience From a Low‐Volume Center,” World Journal of Surgery 48, no. 12 (2024): 2941–2949. 39551645 10.1002/wjs.12402 PMC11619738

[13] Y. Shibayama , N. Wada , H. Umakoshi , et al., “Bilateral Aldosterone Suppression and Its Resolution in Adrenal Vein Sampling of Patients With Primary Aldosteronism: Analysis of Data From the WAVES‐J Study,” Clinical Endocrinology 85, no. 5 (2016): 696–702. 27128234 10.1111/cen.13090

[14] Y. Shibayama , N. Wada , M. Naruse , et al., “The Occurrence of Apparent Bilateral Aldosterone Suppression in Adrenal Vein Sampling for Primary Aldosteronism,” Journal of the Endocrine Society 2, no. 5 (2018): 398–407. 29687091 10.1210/js.2017-00481 PMC5905384

[15] W. Li , Q. Zhou , Y. He , et al., “The Value of LC‐MS/MS in Apparent Bilateral Aldosterone Suppression in Adrenal Venous Sampling for Primary Aldosteronism,” Journal of Clinical Endocrinology & Metabolism 110, no. 9 (2025): e3011–e3020. 39715354 10.1210/clinem/dgae891

[16] T. Wannachalee , P. Vibhatavata , S. Konzen , et al., “Resolution of Paradoxical Bilateral Aldosterone Suppression With Mass Spectrometry,” European Journal of Endocrinology 192, no. 4 (2025): 511–518. 40233185 10.1093/ejendo/lvaf079 PMC12037276

[17] G. Eisenhofer , M. Kurlbaum , M. Peitzsch , et al., “The Saline Infusion Test for Primary Aldosteronism: Implications of Immunoassay Inaccuracy,” The Journal of Clinical Endocrinology & Metabolism 107, no. 5 (2022): e2027–e2036. 34963138 10.1210/clinem/dgab924 PMC9016451

[18] S. Stoner , S. Mogambi , L. Ko , et al., “Investigation of the Possible Cause of Over‐Estimation of Human Aldosterone in Plasma, Using a Unique, Non‐Synthetic Human Aldosterone‐Free Matrix,” Clinical Chemistry and Laboratory Medicine (CCLM) 63 (2025): 2247–2253. 40742120 10.1515/cclm-2024-1468

[19] G. Constantinescu , M. Schulze , M. Peitzsch , et al., “Integration of Artificial Intelligence and Plasma Steroidomics With Laboratory Information Management Systems: Application to Primary Aldosteronism,” Clinical Chemistry and Laboratory Medicine (CCLM) 60 (2022): 1929–1937. 35851438 10.1515/cclm-2022-0470

[20] G. Constantinescu , S. Gruber , S. Fuld , et al., “Steroidomics‐Based Screening for Primary Aldosteronism: Impact of Antihypertensive Drugs,” Hypertension 81, no. 10 (2024): 2060–2071. 39082132 10.1161/HYPERTENSIONAHA.124.23029

[21] M. Peitzsch , T. Dekkers , M. Haase , et al., “An LC‐MS/MS Method for Steroid Profiling During Adrenal Venous Sampling for Investigation of Primary Aldosteronism,” Journal of Steroid Biochemistry and Molecular Biology 145 (2015): 75–84. 25312486 10.1016/j.jsbmb.2014.10.006

[22] F. Alessi , C. Pamporaki , M. Peitzsch , et al., “Mass Spectrometric Measurements of 11‐Deoxycortisol, Androstenedione and Dehydroepiandrosterone Are Superior to Cortisol to Assess Selectivity of Non‐Stimulated Adrenal Vein Sampling,” Clinical Endocrinology (Oxford) 104 (2026): 10–18. 40958155 10.1111/cen.70037 PMC12669813

[23] F. Fanelli , M. Cantù , A. Temchenko , et al., “Report From the Harmoster Study: Impact of Calibration on Comparability of LC‐MS/MS Measurement of Circulating Cortisol, 17OH‐progesterone and Aldosterone,” Clinical Chemistry and Laboratory Medicine (CCLM) 60, no. 5 (2022): 726–739. 35172417 10.1515/cclm-2021-1028

[24] F. Fanelli , S. Bruce , M. Cantù , et al., “Report From the Harmoster Study: Inter‐Laboratory Comparison of LC‐MS/MS Measurements of Corticosterone, 11‐deoxycortisol and Cortisone,” Clinical Chemistry and Laboratory Medicine (CCLM) 61, no. 1 (2023): 67–77. 36288389 10.1515/cclm-2022-0242

[25] F. Fanelli , M. Peitzsch , S. Bruce , et al., “Report From the Harmoster Study: Different LC‐MS/MS Androstenedione, DHEAS and Testosterone Methods Compare Well; However, Unifying Calibration Is a Double‐Edged Sword,” Clinical Chemistry and Laboratory Medicine (CCLM) 62, no. 6 (2024): 1080–1091. 38205643 10.1515/cclm-2023-1138

[26] J. Tate and G. Ward , “Interferences in Immunoassay,” Clinical Biochemist Reviews 25, no. 2 (2004): 105–120. 18458713 PMC1904417

[27] C. M. Sturgeon and A. Viljoen , “Analytical Error and Interference in Immunoassay: Minimizing Risk,” Annals of Clinical Biochemistry: International Journal of Laboratory Medicine 48, no. Pt 5 (2011): 418–432. 10.1258/acb.2011.011073 21750113

[28] G. Ward , A. Simpson , L. Boscato , and P. E. Hickman , “The Investigation of Interferences in Immunoassay,” Clinical Biochemistry 50, no. 18 (2017): 1306–1311. 28847718 10.1016/j.clinbiochem.2017.08.015

[29] K. Ghazal , S. Brabant , D. Prie , and M. L. Piketty , “Hormone Immunoassay Interference: A 2021 Update,” Annals of Laboratory Medicine 42, no. 1 (2022): 3–23. 34374345 10.3343/alm.2022.42.1.3 PMC8368230

[30] M. Wolley , R. D. Gordon , E. Pimenta , et al., “Repeating Adrenal Vein Sampling When Neither Aldosterone/Cortisol Ratio Exceeds Peripheral Yields a High Incidence of Aldosterone‐Producing Adenoma,” Journal of Hypertension 31, no. 10 (2013): 2005–2009. 24107732 10.1097/HJH.0b013e328362add3

[31] M. Siebert , Y. Robert , R. Didier , et al., “Anatomical Variations of the Venous Drainage From the Left Adrenal Gland: An Anatomical Study,” World Journal of Surgery 41, no. 4 (2017): 991–996. 27853815 10.1007/s00268-016-3817-2

[32] K. Omura , H. Ota , Y. Takahashi , et al., “Anatomical Variations of the Right Adrenal Vein: Concordance Between Multidetector Computed Tomography and Catheter Venography,” Hypertension 69, no. 3 (2017): 428–434. 28137990 10.1161/HYPERTENSIONAHA.116.08375

[33] Y. Sato , G. Shirota , K. Makita , et al., “Anatomical Variations of the Left Adrenal Vein Encountered During Venous Sampling,” Journal of Vascular and Interventional Radiology 33, no. 1 (2022): 71–77.e3. 34555539 10.1016/j.jvir.2021.09.005

[34] T. Zelinka , M. Mašek , J. Vlková , et al., “Discrepant Results of Adrenal Venous Sampling in Seven Patients With Primary Aldosteronism,” Kidney and Blood Pressure Research 35, no. 4 (2012): 205–210. 22223126 10.1159/000330720

[35] M. S. Velema , T. Dekkers , A. R. M. M. Hermus , et al., “A Pedunculated Aldosterone‐Producing Adenoma Drained by An Extra Vein Causing Puzzling Results of Adrenal Vein Sampling,” Clinical Endocrinology 89, no. 2 (2018): 242–244. 29741297 10.1111/cen.13736

[36] H. Tannai , Y. Koike , S. Matsui , J. Saito , and K. Makita , “A Rare Independent Left Inferior Phrenic Vein Sampling in a Left Adrenal Aldosterone‐Producing Adenoma,” Radiology Case Reports 16, no. 6 (2021): 1443–1446. 33912260 10.1016/j.radcr.2021.03.012 PMC8065199

[37] J. Yu , C. Fan , W. Wei , et al., “Duplicated Adrenal Veins in Primary Aldosteronism Misdiagnosed With Ectopic Aldosteronoma Due to Apparent Bilateral Aldosterone Suppression,” Blood Pressure 32, no. 1 (2023): 2209664. 37183447 10.1080/08037051.2023.2209664

[38] S. Miyamoto , Y. Yoshida , S. Miyamoto , H. Nishida , Y. Asayama , and H. Shibata , “Segmental Adrenal Venous Sampling in Unilateral Primary Aldosteronism With Apparent Bilateral Aldosterone Suppression,” JCEM Case Reports 2, no. 9 (2024): luae164. 39286517 10.1210/jcemcr/luae164 PMC11403205

[39] S. Y. T. Tan , K. S. Ng , C. Tan , M. Chuah , M. Zhang , and T. H. Puar , “Bilateral Aldosterone Suppression in Patients With Right Unilateral Primary Aldosteronism and Review of the Literature,” Journal of the Endocrine Society 4, no. 4 (2020): bvaa033. 32285021 10.1210/jendso/bvaa033 PMC7138278

[40] X. Wu , R. Senanayake , E. Goodchild , et al., “[(11)C]metomidate PET‐CT Versus Adrenal Vein Sampling for Diagnosing Surgically Curable Primary Aldosteronism: A Prospective, Within‐Patient Trial,” Nature Medicine 29, no. 1 (2023): 190–202. 10.1038/s41591-022-02114-5 PMC9873572 36646800

[41] K. Wang , H. Cong , Z. Gao , et al., “An Innovative Immunoassay for Accurate Aldosterone Quantification: Overcoming Low‐Level Inaccuracy and Renal Dysfunction‐Associated Interference,” Clinical Chemistry and Laboratory Medicine (CCLM) 63, no. 12 (2025): 2477–2484. 40842291 10.1515/cclm-2025-0743

[42] J. Zhang , B. Luo , Z. Fan , et al., “Liquid Chromatography‐Tandem Mass Spectrometry‐Based Recalibration Reduces Inter‐Platform Variability in Aldosterone Detection Across Chemiluminescence Immunoassay Platforms,” Clinica Chimica Acta 579 (2025): 120604. 10.1016/j.cca.2025.120604 41101439

