# Supplementary material for: Apparent Bilateral Aldosterone Suppression During Adrenal Vein Sampling: An Artefact of the Liaison Chemiluminescence Immunoassay and Other Causes Revealed by Comparisons With Another Commercial Immunoassay and Mass Spectrometry
Source: Clin Endocrinol (Oxf). 2026 Mar 29;105(2):175–84. doi: 10.1111/cen.70135 (PMC13326992; doi:10.1111/cen.70135)
Supplement: Supplementary file 1 — Supplement‐Bilateral‐suppression‐26122025. [file CEN-105-175-s001.pdf]

Apparent bilateral aldosterone suppression during adrenal vein sampling: an artefact of the Liaison chemiluminescence immunoassay and other causes revealed by comparisons with another commercial immunoassay and mass spectrometry

Christina Pamporaki[1], Carmina T Fuss[2], Ralph Kickuth[3], Lydia Kürzinger[2], Mirko Peitzsch[4], Sybille Fuld[1], Manuel Schulze[5], Jun Yang[6], Martin Reincke[7], Sven Gruber,[8] Felix Beuschlein[7,8], Jacques W.M. Lenders[9] and Graeme Eisenhofer[1]

[1]Department of Medicine III, University Hospital Carl Gustav Carus, Technische Universität Dresden, Germany; [2]Department of Internal Medicine I, Division of Endocrinology and Diabetes and [3]Department of Radiology, University Hospital, University of Würzburg, Germany; [4]Institute of Clinical Chemistry and Laboratory Medicine, Medical Faculty and University Hospital Carl Gustav Carus, Technische Universität Dresden, Germany; [5]Center for Interdisciplinary Digital Sciences, Department Information Services and High Performance Computing, Technische Universität Dresden, Germany; [6]Centre for Endocrinology and Metabolism, Hudson Institute of Medical Research, Clayton, Australia; [7]Department of Medicine IV, University Hospital, Ludwig Maximilian University Munich, Munich; [8]Department of Endocrinology, Diabetology and Clinical Nutrition, University Hospital Zurich and the LOOP Zurich Medical Research Center, Zurich, Switzerland; [9]Department of Internal Medicine, Radboud University Medical Center, Nijmegen, the Netherlands.

Contents

Section	Page
Patient recruitment	2
Study design and patient flow	2
Supplemental figure 1	3
Bilateral selectivity	4
Supplemental table 1	4
Adrenal and peripheral venous plasma aldosterone and cortisols	5
Supplemental table 2	5
RASI values after replacement of immunoassay with LC-MS/MS measured cortisol	6
Supplemental figure 2	6
Supplemental figure 3	6
Passing Bablok and Bland Altman analyses	7
Supplemental figure 4	7
Supplemental figure 5	8
Sources of immunoassay interference *(included as a reviewer response)*	9
References	10

Patient recruitment

PROSALDO (the **PRO**spective **S**tudy on the diagnostic value of steroid profiling in primary **ALDO**steronism) is a registered international multicentre trial (trial registration no: DRKS00017084 https://drks.de/search/en/trial/DRKS00017084). Patients were recruited into the study at seven tertiary care centres: University Hospitals Dresden, Würzburg and Munich in Germany; University Hospital Zurich in Switzerland; Hudson Institute of Medical Research, Clayton, Victoria and Prince of Wales and St George Hospitals, Sydney, Australia.

Inclusion into the protocol required suspicion of PA based on hypertension and at least one of several other criteria: 1. office blood pressure above 150/100 mmHg on two separate visits; 2. therapy resistant hypertension with at least 3 different antihypertensives, including a diuretic; 3. spontaneous or diuretic-induced hypokalemia; 4. an adrenal incidentaloma; 5. family history of PA, early onset hypertension or cerebrovascular accident (<40 years age); or 6. obstructive sleep apnea.

Patients were excluded at recruitment based on several criteria: 1. presence of other forms of secondary hypertension; 2. necessity for continued use of medications that interfere with laboratory test results; 3. established low plasma concentrations of aldosterone (<170 pmol/L by immunoassay measurements); 4. pregnancy; 5. impaired mental capacity that precluded informed consent; 5. glucocorticoid remedial hyperaldosteronism; and 6. severe or terminal co-morbidity that precluded required investigational procedures and/or therapeutic interventions.

Study design, procedures and patient flow

The study was designed according to a primary objective, which was to evaluate the combination of mass spectrometry-based steroid profiling and machine learning for the diagnostic stratification of patients with suspected primary aldosteronism. Patient recruitment began in January 2019. Requirements for the primary objective were satisfied at the end of 2023. Nevertheless, recruitment of patients continued to satisfy secondary study objectives. One of several secondary objectives of the PROSALDO trial was to examine utility of liquid chromatography with tandem mass spectrometry (LC-MS/MS) for measurements of adrenal-derived steroids during adrenal venous sampling (AVS). It was hypothesised that these measurements would offer diagnostic advantages over routine measurements of aldosterone and cortisol. The presently reported study takes advantage of those LC-MS/MS-derived measurements for comparison of immunoassay-based measurements of aldosterone and cortisol during AVS.

Details about the protocol and the flow of patients through the study have been described in several other manuscripts [1-6]. Those details are again repeated here. The general flow of patients through the study followed recommendations of the 2016 Endocrine Society guideline [7]. Initial screening required two or more measurements of the aldosterone-to-renin ratio (ARR) carried out on separate days. One measurement was required when patients were not taking antihypertensive medications that interfere with measurements of aldosterone or renin. If required, antihypertensive therapy was adjusted to an alpha-adrenoceptor blocker and/or a non-dihydropyridine calcium channel blocker. Patients in whom adjustments to medications were not possible were excluded from this requirement if directional differences in the ARR relative to known impacts of interfering drugs were not compatible with established influences to cause false-positive or a false-negative results.

In addition to routine measurements of aldosterone and renin, samples of plasma were also collected at each phase of the protocol for LC-MS/MS measurements of steroid profiles. Those samples were shipped to the Dresden laboratory where LC-MS/MS measurements were performed. Collection of all required data for the PROSALDO protocol was facilitated with electronic case report forms (eCRFs), as supported by a research electronic data capture (REDCap) tool [8, 9]. The REDCap system also

facilitated automated generation of reports that included steroid profiles, associated reference intervals, and machine learning-based interpretations of steroid profiles. Investigators at study centres were provided with reports as PDF files after review by the study coordinator and staff at the Dresden laboratory responsible for LC-MS/MS measurements. The protocol thereby involved use of steroid profile-derived machine learning-probability scores as aa additional and separate tool from the ARR for purposes of screening (Supplemental figure 1). The steroid profiles enabled identification of some patients with primary aldosteronism who had false-negative test results for the ARR.

The seated saline suppression test (SSST) was employed to follow up on positive results at screening for either or both the ARR and steroid profile-derived probability scores (Supplemental figure 1). A positive result for the SSST was initially defined according to post-infusion plasma concentrations of aldosterone above 170 pmol/L according to measurements performed locally at each study centre. Additional interpretation of test results was based on measurements of aldosterone by LC-MS/MS at Dresden. The cut-off for a positive test result was set at ≥162 pmol/L, as defined by Thuzar and colleagues in 2020 for LC-MS/MS measurements of aldosterone [10]. The SSST was further validated for LC-MS/MS measurements of aldosterone in 420 patients of the PROSALDO trial [6]. An optimal cut-off was defined at 169 pmol/L, close to that originally defined by Thuzar et al. [10]. That cut-off allowed for sufficient sensitivity and specificity (96% each) for disease confirmation and exclusion provided that standard operating procedures were appropriately followed.

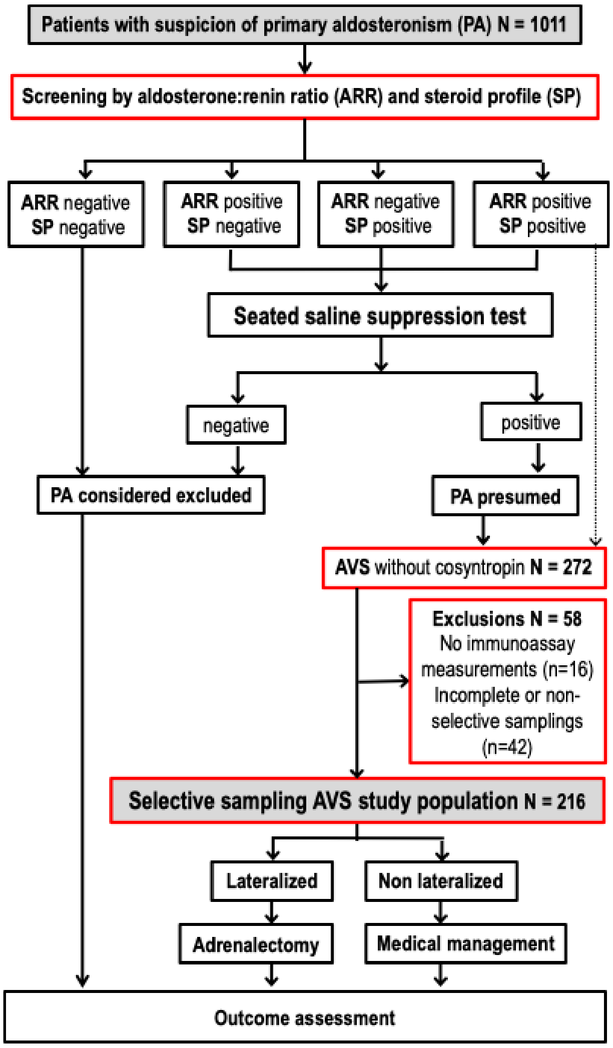

Supplemental figure 1. Patient flow though the study from initial recruitment to final outcome assessment. The present report focuses on a single phase (AVS subtyping) in the flow of patients through the protocol.

Flow of patients through the protocol to AVS required positive results for one or both screening tests and a positive result for the SSST (Supplemental figure 1). Exceptions included some patients who proceeded directly from screening to AVS based on positive screening test results that were unlikely to be false-positive and where the presentation was strongly consistent with a diagnosis of primary aldosteronism. During the first few years of the PROSALDO trial (2019-2022), before immunoassay inaccuracy was identified to result in high rates of false-positive test results for the SSST, flow of patients to AVS was based on immunoassay measurements of aldosterone. For some centres this persisted where there was perceived need for continued reliance on certified immunoassays. Progression to AVS also required willingness of patients to undergo adrenalectomy according to results of subtyping, including imaging studies.

Outcome assessments of patients who underwent adrenalectomy were carried out six or more months after surgical intervention to determine presence of unilateral primary aldosteronism according to biochemical and/or clinical remission [11]. Immunohistochemical staining to confirm presence of aldosterone synthase was also carried out according to international consensus criteria (HISTALDO), which provided further evidence that excised adrenals contained aldosterone producing lesions [12]. Furthermore, the PROSALDO protocol stipulated that all other patients undergo follow-up outcome assessments at six months or more (usually a year to two years) after the last test or intervention to confirm previous negative or positive evidence of primary aldosteronism (Supplemental figure 1).

Bilateral selectivity

Bilateral selectivity was assessed according to measurements of four adrenal steroids (cortisol, 11-deoxycortisol, androstenedione and DHEA) as described elsewhere [13]. Among the 22 patients with apparent bilateral aldosterone suppression there were 21 in who selectivity indices for all four steroids ≥2 (Supplemental table 1). The one exception was a patient (#16) with selectivity indices for cortisol in the right adrenal vein of 1.3 and 1.4 by immunoassay and LC-MS/MS measurements. However, that patient showed selective sampling according to the three other measures, which as described elsewhere are superior to cortisol to assess selective sampling [13].

Supplemental table 1. Selectivity indices for right and left adrenal vein sampling sites for the 22 patients with apparent bilateral aldosterone suppression

Immunoassay				LC-MS/MS							
		Cortisol		Cortisol		11-Deoxycortisol		Androstenedione		DHEA	
	Method	Right	Left	Right	Left	Right	Left	Right	Left	Right	Left
1	Elecsys	18.9	29.4	8.5	11.6	34.1	48.6	51.2	65.2	92.4	80.1
2	Elecsys	70.1	127.1	47.3	58.7	255.8	334.0	258.0	327.6	366.7	593.9
3	Elecsys	97.0	105.6	106.0	92.6	348.5	328.1	455.1	406.5	1043.6	1114.9
4	Elecsys	9.9	14.4	8.7	12.5	36.6	54.5	56.6	93.4	27.5	33.1
5	Liaison	104.7	51.2	42.1	20.0	416.1	140.9	154.4	107.2	273.3	126.5
6	Liaison	114.0	98.1	50.6	51.5	304.4	258.0	333.3	419.5	532.4	587.1
7	Elecsys	6.2	7.6	5.9	6.8	22.9	27.2	36.7	54.9	43.5	41.9
8	Elecsys	15.0	4.7	13.2	4.6	40.4	14.5	96.8	21.3	87.0	16.7
9	Beckman	38.2	20.1	7.3	11.1	24.3	20.7	34.1	32.6	37.9	31.8
10	Elecsys	11.7	15.7	10.8	14.4	33.2	50.5	41.2	63.5	37.6	44.5
11	Liaison	24.5	12.7	5.9	6.6	13.9	14.0	20.0	26.5	12.9	11.1
12	Liaison	15.5	55.8	6.8	26.3	27.1	115.1	70.4	141.5	111.4	196.3
13	Liaison	51.5	46.5	26.4	24.3	194.9	171.5	138.1	158.5	155.7	123.1
14	Elecsys	62.1	42.4	38.5	30.5	67.1	75.5	82.8	94.6	132.9	101.3
15	Liaison	67.3	41.3	28.0	18.0	79.3	49.2	95.2	38.3	110.9	30.0
16	Elecsys	1.3	33.5	1.4	29.0	2.0	93.4	2.3	127.4	3.2	167.8
17	Elecsys	29.9	40.0	29.6	38.8	208.4	241.2	111.1	208.4	113.0	211.1
18	Elecsys	29.1	2.4	29.3	2.1	332.4	16.8	160.2	23.0	155.1	17.1
19	Elecsys	2.8	49.3	2.4	65.3	6.2	374.2	3.2	282.2	3.0	291.9
20	Elecsys	26.1	10.8	23.7	14.1	119.8	46.5	64.0	34.6	246.4	71.3
21	Elecsys	3.0	13.9	6.1	27.2	10.0	61.8	28.8	55.6	23.0	43.7
22	Elecsys	2.3	3.1	3.3	4.3	50.0	79.3	31.0	51.0	36.0	48.6

Adrenal and peripheral venous plasma aldosterone and cortisol

Among the 22 patients with apparent bilateral aldosterone suppression, there was considerable variation in plasma concentrations of aldosterone measured in right and left adrenal venous plasma compared to peripheral venous plasma for both immunoassay and LC-MS/MS measurements (Supplemental table 2). Plasma concentrations of aldosterone at peripheral venous sampling sites were 118% higher (P<0.0001) by immunoassay than by LC-MS/MS measurements, but showed no differences between immunoassay and LC-MS/MS measurements at adrenal venous sampling sites. Plasma cortisol was measured at 86% and 59% higher (P<0.0001) concentrations in respective right and left adrenal veins by immunoassay than by LC-MS/MS, compared to only 14% higher (P=0.0487) concentrations in peripheral veins.

Supplemental table 2. Plasma concentrations of aldosterone and cortisol by immunoassay and LC-MS/MS measurements in right and left adrenal veins (RV & LV) versus a peripheral vein (PV) in patients with apparent bilateral aldosterone suppression

	Immunoassay								LC-MS/MS					
	Aldosterone (pmol/L)				Cortisol (nmol/L)				Aldosterone (pmol/L)			Cortisol (nmol/L)		
	Method	RV	LV	PV	Method	RV	LV	PV	RV	LV	PV	RV	LV	PV
1	Liaison	3579	4716	433	Elecsys	9356	14542	494	4231	4730	266	3887	5315	459
2	Liaison	34680	58262	1165	Elecsys	16754	30368	239	138844	91666	740	13138	16277	277
3	Liaison	2605	70886	968	Elecsys	44235	48154	456	1404	84008	461	36712	32070	346
4	Liaison	2336	3568	383	Elecsys	2269	3299	229	2996	5368	79	2123	3063	244
5	Liaison	8240	3829	308	Liaison	21371	10445	204	7863	4278	186	11877	5660	282
6	Liaison	6964	9045	302	Liaison	50334	43282	441	7507	10249	55	20150	20511	399
7	Liaison	319	1190	308	Elecsys	2315	2840	376	83	929	86	2406	2769	409
8*	Liaison	1845	591	585	Elecsys	11076	3476	739	1470	361	341	8007	2751	605
9	Liaison	2540	676	387	Beckmar	5615	2951	147	3843	486	266	1044	1594	143
10	Liaison	45167	7602	6409	Elecsys	2786	3746	238	50924	4564	2830	2550	3394	235
11†	Liaison	4883	791	333	Liaison	7030	3785	292	3335	727	182	1670	1830	280
12	Liaison	1948	5882	178	Liaison	8062	28952	519	2064	5826	75	2649	10165	387
13	Liaison	11652	12707	314	Liaison	36390	32826	706	14427	11944	172	13508	12434	512
14	Liaison	40783	33015	918	Elecsys	30343	20722	489	44057	39854	551	18970	15015	493
15	Liaison	5632	5687	222	Liaison	21354	13110	317	4173	4994	103	8378	5391	299
16	Liaison	583	10959	469	Elecsys	489	12583	373	375	15537	280	513	10547	364
17*	iSYS	3446	6983	219	Elecsys	8001	10705	268	3399	5160	175	8576	11242	290
18*	iSYS	2358	541	719	Elecsys	11477	941	395	1526	194	563	11001	796	376
19	iSYS	763	12607	616	Elecsys	692	12388	251	178	8789	424	460	12591	193
20*	iSYS	2053	1958	935	Elecsys	9152	4497	384	1510	1644	705	7454	6210	315
21*	iSYS	2999	4433	2092	Elecsys	1661	7670	552	1143	1604	996	1605	7159	263
22**	iSYS	907	355	225	Elecsys	993	1355	441	264	83	86	954	1248	292

* Patients with evidence of bilateral aldosterone suppression by both immunoassay and LC-MS/MS measurements. **Patient 21 is the sole patient with bilateral suppression by mass spectrometry and not by immunoassay measurements. †This patient underwent sequential sampling and results for the PV are presented as mean values of two sampling time points for immunoassay measurements of aldosterone (327 & 328 pmol/L) and cortisol (287 & 298 nmol/L) as well as mass spectrometric measurements of aldosterone (287 & 298 pmol/L) and cortisol (282 & 277 nmol/L).

RASI values after replacement of immunoassay with LC-MS/MS measured cortisol

Among all 216 patients, there were 17 (7.9%) with apparent bilateral aldosterone suppression after replacement of immunoassay measured cortisol with LC-MS/MS measured cortisol in RASI values calculated with immunoassay measurements of aldosterone (Supplemental figure 2). Compared to the 21 patients in whom immunoassay measurements had previously suggested bilateral aldosterone suppression (Figure 1 and table 1 of the main manuscript), this represented a reduction of four patients with apparent bilateral aldosterone suppression, all involving patients with measurements of aldosterone by the Liaison immunoassay. Nevertheless, the prevalence of apparent bilateral aldosterone suppression, at 7.9% with immunoassay measurements of aldosterone, remained higher (P=0.0037) than the prevalence of 3.2% with LC-MS/MS measurements of aldosterone. The prevalence of apparent bilateral suppression with the Liaison immunoassay of aldosterone, at 10.9%, remained higher (P=0.0007) than the prevalence of 0.9% with LC-MS/MS measurements. In contrast, the prevalence of apparent bilateral aldosterone suppression did not differ between measurements of aldosterone by the iSYS immunoassay compared to LC-MS/MS (4.7% versus 5.7%). These observations indicate that apparent bilateral suppression of aldosterone with immunoassay measurements largely reflects an artefact of the Liaison immunoassay of aldosterone with minimal impact of cortisol.

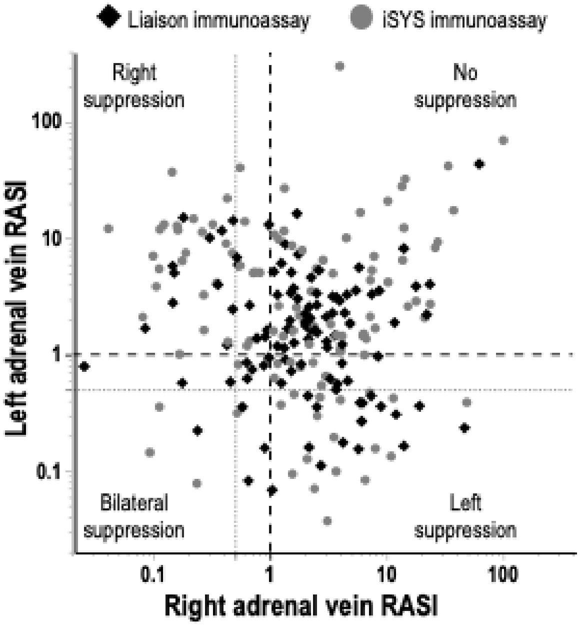

Supplemental figure 2. Scatterplot relationship of RASI values based on Liaison and iSYS immunoassay measurements of aldosterone after replacement of immunoassay measurements with LC-MS/MS measurements of cortisol.

Scatterplot relationships RASI values calculated from LC-MS/MS measurements of both aldosterone and cortisol versus those calculated using immunoassay measurements of aldosterone and LC-MS/MS measurements of cortisol showed strong positive relationships (P<0.0001) for both right and left adrenal vein samplings (Supplemental figure 3). Relationships followed lines of identity for RASI values calculated using the iSYS CLIA for aldosterone, but showed a rightward shift away from lines of identity to lower RASI values for Liaison CLIA-based measurements of aldosterone.

Supplemental figure 3. Scatterplot relationships of RASI values calculated for right (A) and left (B) adrenal vein samplings based on LC-MS/MS measurements of both aldosterone and cortisol (x-axes) versus immunoassay measurements of aldosterone and LC-MS/MS measurements of cortisol. Relationships are shown relative to lines of identity.

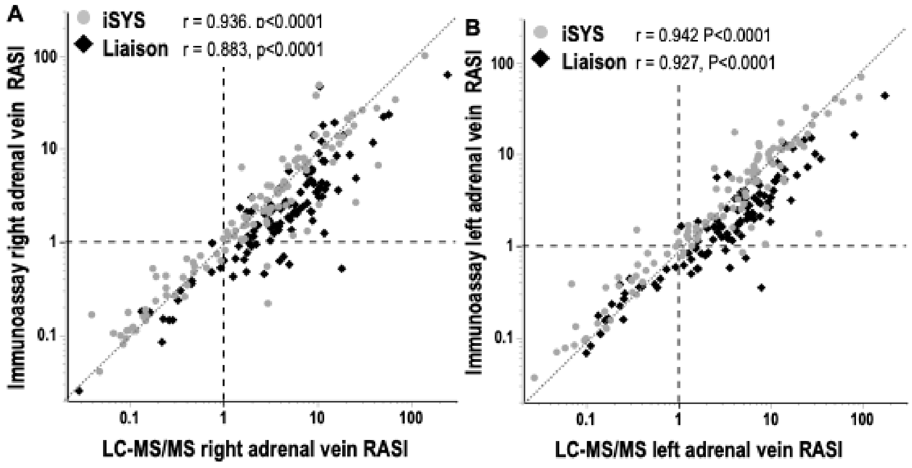

Passing Bablok and Bland Altman analyses

Comparisons of plasma aldosterone as measured by the iSYS CLIA and Liaison CLIA showed divergent differences with measurements by LC-MS/MS according to both Passing Bablok and Bland Altman analyses (Supplemental figure 4). Passing Bablok analyses indicated strong positive relationships (P<0.0001) of immunoassay and LC-MS/MS measured plasma aldosterone for both Liaison and iSYS CLIAs (Supplemental figure 4A&B). Bland-Altman analyses indicated similarly minimal 28% and 39% higher (P<0.0001) measurements of aldosterone by respective Liaison and iSYS immunoassays compared to LC-MS/MS (Supplemental figure 4C&D). However, those differences showed divergences at low and high plasma concentrations. Plasma concentrations of aldosterone measured by the iSYS CLIA were higher than by LC-MS/MS over all concentration ranges. In contrast, higher concentrations measured by the Liaison CLIA compared to LC-MS/MS were only manifest at lower concentrations.

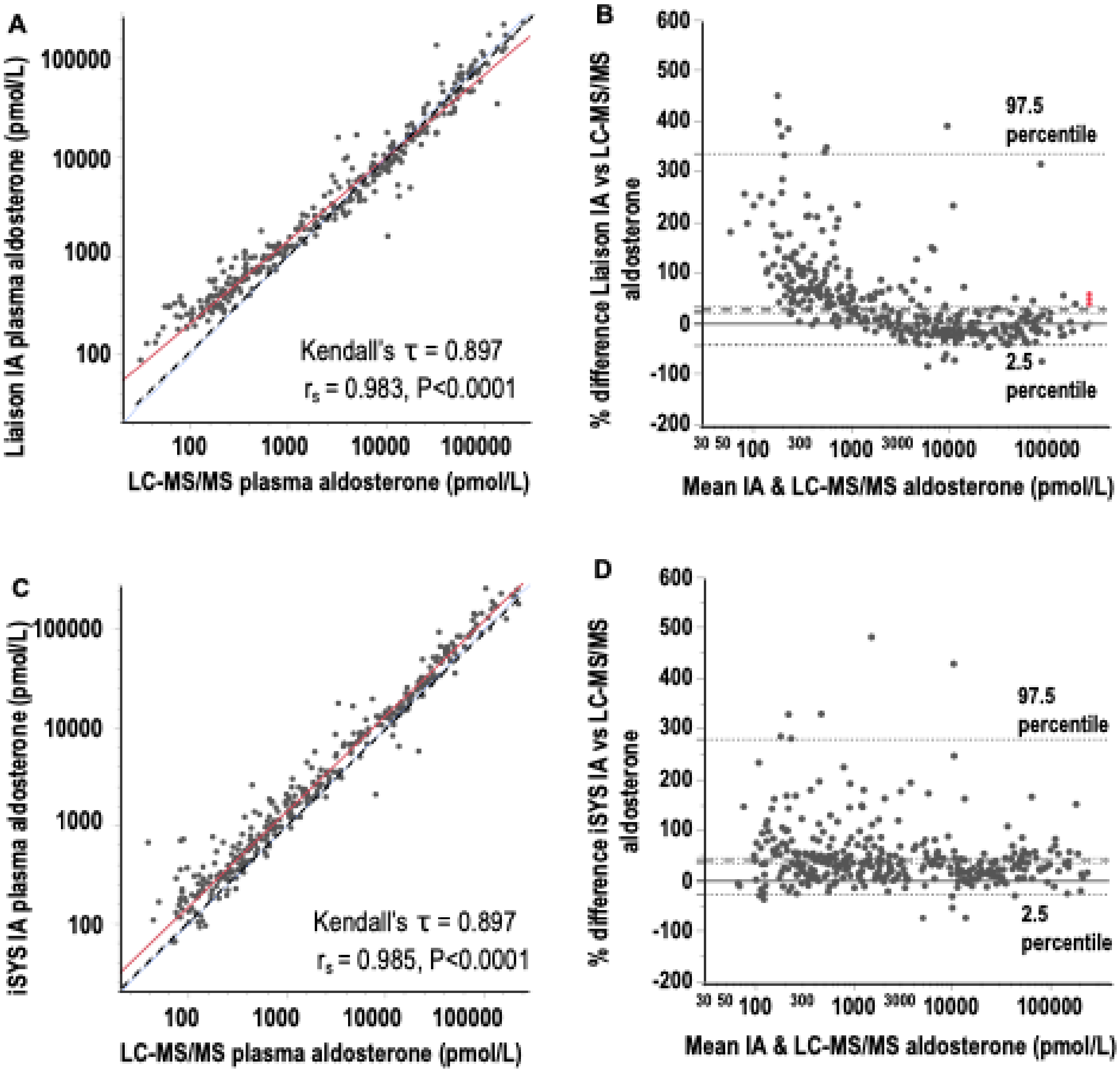

Supplemental figure 4. Passing Bablok relationships and Bland Altman plots (for Liaison immunoassay (A,B) and ISYS immunoassay (C,D) based measurements of aldosterone. Dashed lines in Passing-Bablok displays (A,C) indicate lines of identity, whereas solid lines indicate best fits of relationships. For Bland-Altman plots (B,D) dashed lines indicate geometric means for all data across the entire concentration range, which are bounded closely by 95% confidence intervals and more widely by the 97.5 and 2.5 percentiles.

Respective Passing Bablok and Bland-Altman analyses of relationships and differences in measurements of cortisol by the three different immunoassays compared to LC-MS/MS revealed largely strong (P<0.0001) positive relationships and variable differences in plasma concentrations measured by immunoassays compared to LC-MS/MS (Supplemental figure 4). Although all three immunoassays returned higher (P<0.0001) measured concentrations of cortisol than indicated by LC-MS/MS, the extent of these differences varied according to the immunoassay. Among the three immunoassays,

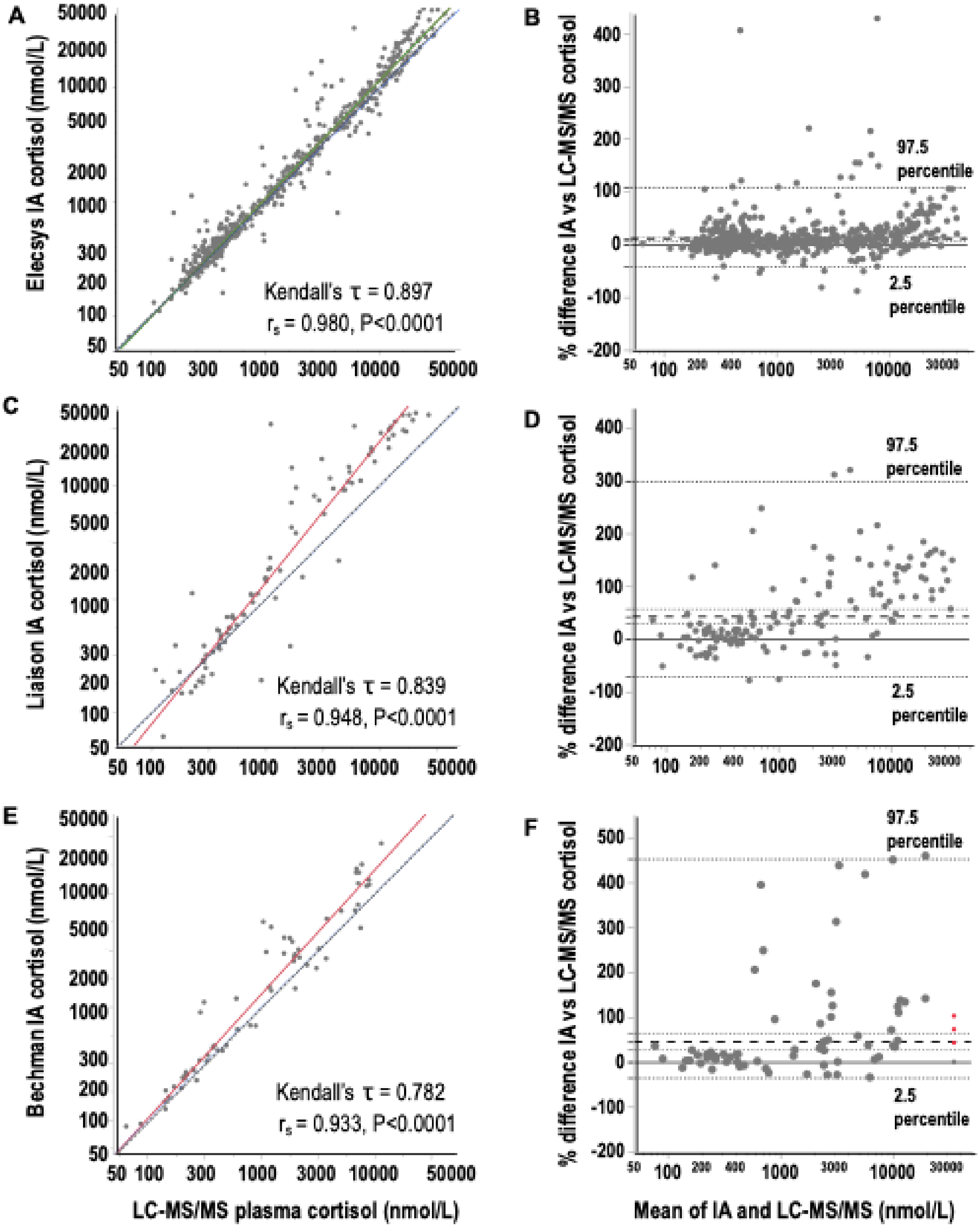

Supplemental figure 5. Passing Bablok and Bland-Altman analyses for measurements of cortisol by the Elecsys (A&B), Liaison (B&C) and Bechman immunoassays (IA) compared to LC-MS/MS. Lines of identity for Passing-Bablok analyses (A,C,D) are shown by dotted lines, whereas biases in measurements indicated by Bland-Altman analyses (B,C,F) are shown by dashed lines bounded by confidence intervals.

measurements of cortisol by the Elecsys immunoassay displayed the closest agreement with LC-MS/MS according to concentrations that were only 10.3% (CI 7.6-12.9%) higher than those measured by LC-MS/MS. In contrast, measured concentrations of cortisol by Liaison and Bechman immunoassays were respectively 43.4% (CI 30.8-57.2%) and 45.7% (CI 27.7-66.2%) higher than plasma concentrations measured by LC-MS/MS. The extent of scatter was also more considerable and correlation coefficients weaker for relationships of those two immunoassays with LC-MS/MS measurements compared to the Elecsys immunoassay of cortisol.

Sources of immunoassay interferences

Macromolecular interferences with commercial Liaison and iSYS chemiluminescence immunoassays were first identified according to immunoassay measurements of plasma aldosterone with and without a solid phase sample extraction/purification step applied to three aliquots of a plasma specimen [2]. That extraction/purification step employed the same procedure used for LC-MS/MS measurements. The procedure removes proteins and other macromolecules, but retains lower molecular weight analytes, including steroids. Thus, the extracted sample contains steroids but not proteins and other macromolecules. Plasma concentrations of aldosterone measured by the immunoassays in extracted plasma specimens were substantially reduced compared to measurements without sample extraction and similar to concentrations measured by LC-MS/MS in aliquots of the same plasma specimens. These findings were confirmed for the Liaison chemiluminescence immunoassay in a subsequent study that employed dichloromethane for sample purification [14]. Those two independent studies confirm that inaccuracies of the Liaison and iSYS chemiluminescence immunoassays for aldosterone are due to interferences from circulating macromolecules. The specific macromolecules responsible for the interference remain unidentified.

Proteins are the most likely source of macromolecular interference since these comprise close to 90% of all circulating macromolecules. Lipids associated with lipoproteins, which represent at most 10% of the remaining macromolecular mass, are less likely than proteins to be the source of interference due to their nature and common structure. Carbohydrate glycoconjugates exist in plasma largely bound to proteins, while nucleic acids are only present in trace amounts and have not been identified to interfere with immunoassays. Among circulating proteins, albumin predominates over the (55-60%) the globulins (35-40%). Proteins within the globulin pool include binding alpha globulins, transport and complement beta globulins and gamma globulins, primarily immunoglobulins. Members of the globulin pool are well established to interfere with immunoassays.

Although the specific proteins that are responsible for the interferences with Liaison and iSYS immunoassays of aldosterone have not been identified, there are potential clues from past studies of interferences with other immunoassays, as covered in several reviews [15-18]. In the review by Tate and Ward [15], sex-hormone binding globulin was described to interfere with radioimmunoassays of testosterone and estradiol that did not employ a sample purification step. Interferences by auto-analyte antibodies have also been described, but such interferences mainly impact higher molecular weight analytes, such as thyroglobulin, insulin and prolactin. Patients with autoimmune disorders are most vulnerable to these forms of immunoassay interference. Auto-analyte antibodies are also less likely to be responsible for interferences with lower molecular weight analytes, haptens, that are too small alone to elicit an antigenic response.

Heterophile antibodies are another source of interference with immunoassays [15-18]. Interference is by non-competitive mechanisms, usually involving binding to components of the immunoassay detection systems. Rheumatoid factors, predominantly of the immunoglobulin M isotype, represent another source of immunoassay innaccuracy with a similar non-competitive mechanism of interference

to heterophile antibodies. This source of interference in most common in patients with rheumatoid arthritis or other autoimmune diseases.

Other related and relevant sources of immunoassay interference include human anti-animal antibodies [15-18]. These are antibodies produced against specific animal immunoglobulins, such as from mice, rats, rabbits, goats, sheep and other animals that humans may come into contact with. If present within a patient, such anti-animal antibodies can interfere with immunoassays that employ antibodies from that particular animal. Antibodies generated against streptavidin can represent a significant cause of interference for immunoassays that incorporate streptavidin-biotin technology. Streptavidin is produced by a specific soil microorganism, *Streptomyces avidinii*. Antibodies to streptavidin in some patients may arise from contact to that micro-organism and if present may interfere with the streptavidin-biotin complex involved in generating fluorescence or other signals. Presence in serum or plasma of biotin, now often used as a health supplement, can also similarly interfere with immunoassays that employ streptavidin-biotin technology.

All aforementioned sources of interference are particularly more troublesome with competitive than non-competitive immunoassays. The former immunoassays are the most commonly employed for haptens, such as steroids. This is because generation of antibodies for use in immunoassays requires conjugation of the hapten to a higher molecular weight molecule in order to generate an antigenic response. The assay architecture thereafter employed is dictated by need to include the hapten-conjugate within the immunoassay detection and signalling system. Differing immunoassay architecture, including employed antibodies, impacts susceptibility to different types on interferences.

The preceding considerations highlight that interferences must be expected to vary from immunoassay to immunoassay and also among different individuals for the same immunoassay. Those expectations have been confirmed for the Liaison and iSYS immunoassays for aldosterone [2]. As detailed in that manuscript, assays of aliquots of identical plasma samples from specific patients were found to be substantially overestimated compared to LC-MS/MS measurements for one immunoassay and not the other. Interferences were also far more prominent for the Liaison than the iSYS chemiluminescence immunoassay. Moreover, immunoassay measurements of aldosterone in samples collected from the same patients on different days were shown to show similar extents of overestimation inaccuracy. Thus, interference-associated overestimation inaccuracy varies among patients from repeatedly negligible or minor overestimation for some patients to repeatedly extensive overestimation for other patients. Such inaccuracies are critically important for the understanding of clinicians who use the results of these immunoassays for management of their patients.

References

1. Constantinescu G, Bidlingmaier M, Gruber M, et al. Mass spectrometry reveals misdiagnosis of primary aldosteronism with scheduling for adrenalectomy due to immunoassay interference. Clin Chim Acta. 2020;507:98-103.
2. Eisenhofer G, Kurlbaum M, Peitzsch M, et al. The Saline Infusion Test for Primary Aldosteronism: Implications of Immunoassay Inaccuracy. J Clin Endocrinol Metab. 2022;107(5):e2027-e2036.
3. Constantinescu G, Schulze M, Peitzsch M, et al. Integration of artificial intelligence and plasma steroidomics with laboratory information management systems: application to primary aldosteronism. Clin Chem Lab Med. 2022;60:1929-1937.
4. Fuld S, Constantinescu G, Pamporaki C, et al. Screening for Primary Aldosteronism by Mass Spectrometry Versus Immunoassay Measurements of Aldosterone: A Prospective Within-Patient Study. J Appl Lab Med. 2024;9(4):752-766.
5. Constantinescu G, Gruber S, Fuld S, et al. Steroidomics-Based Screening for Primary Aldosteronism: Impact of antihypertensive Drugs. Hypertension. 2024;81(10):2060-2071.

6. Pamporaki C, Remde H, Constantinescu G, et al. The saline infusion test with mass spectrometric measurements of aldosterone to confirm primary aldosteronism. J Hypertens. 2025;“in press”.
7. Funder JW, Carey RM, Mantero F, et al. The management of primary aldosteronism: case detection, diagnosis, and treatment: An Endocrine Society Clinical Practice Guideline. J Clin Endocrinol Metab. 2016;101:1889-1916.
8. Harris PA, Taylor R, Thielke R, et al. Research electronic data capture (REDCap)--a metadata-driven methodology and workflow process for providing translational research informatics support. J Biomed Inform. 2009;42(2):377-381.
9. Harris PA, Taylor R, Minor BL, et al. The REDCap consortium: Building an international community of software platform partners. J Biomed Inform. 2019;95:103208.
10. Thuzar M, Young K, Ahmed AH, et al. Diagnosis of Primary Aldosteronism by Seated Saline Suppression Test-Variability Between Immunoassay and HPLC-MS/MS. J Clin Endocrinol Metab. 2020;105(3):e477-e483.
11. Williams TA, Lenders JWM, Mulatero P, et al. Outcomes after adrenalectomy for unilateral primary aldosteronism: an international consensus on outcome measures and analysis of remission rates in an international cohort. Lancet Diabetes Endocrinol. 2017;5(9):689-699.
12. Williams TA, Gomez-Sanchez CE, Rainey WE, et al. International Histopathology Consensus for Unilateral Primary Aldosteronism. J Clin Endocrinol Metab. 2021;106(1):42-54.
13. Alessi F, Pamporaki C, Peitzsch M, et al. Mass Spectrometric Measurements of 11-Deoxycortisol, Androstenedione and Dehydroepiandrosterone Are Superior to Cortisol to Assess Selectivity of Non-Stimulated Adrenal Vein Sampling. Clin Endocrinol (Oxf). 2025.
14. Stoner S, Mogambi S, Ko L, et al. Investigation of the possible cause of over-estimation of human aldosterone in plasma, using a unique, non-synthetic human aldosterone-free matrix. Clin Chem Lab Med. 2025;63(11):2247-2253.
15. Tate J, Ward G. Interferences in immunoassay. Clin Biochem Rev. 2004;25(2):105-120.
16. Sturgeon CM, Viljoen A. Analytical error and interference in immunoassay: minimizing risk. Ann Clin Biochem. 2011;48(Pt 5):418-432.
17. Ward G, Simpson A, Boscato L, et al. The investigation of interferences in immunoassay. Clin Biochem. 2017;50(18):1306-1311.
18. Ghazal K, Brabant S, Prie D, et al. Hormone Immunoassay Interference: A 2021 Update. Ann Lab Med. 2022;42(1):3-23.
